# Histone Arginine Methyltransferase CARM1-Mediated H3R26me2 Is Essential for Morula-to-Blastocyst Transition in Pigs

**DOI:** 10.3389/fcell.2021.678282

**Published:** 2021-06-02

**Authors:** Zubing Cao, Xu Tong, Huiqun Yin, Naru Zhou, Xiangdong Zhang, Mengya Zhang, Xin Wang, Qiuchen Liu, Yelian Yan, Yangyang Ma, Tong Yu, Yunsheng Li, Yunhai Zhang

**Affiliations:** ^1^Anhui Province Key Laboratory of Local Livestock and Poultry, Genetical Resource Conservation and Breeding, College of Animal Science and Technology, Anhui Agricultural University, Hefei, China; ^2^Reproductive Medicine Center, The 901st Hospital, Hefei, China; ^3^Reproductive and Genetic Branch, The First Affiliated Hospital of University of Science and Technology of China, Hefei, China

**Keywords:** CARM1, H3R26me2, blastocyst, pig, lineage specification

## Abstract

Coactivator-associated arginine methyltransferase 1 (CARM1) is involved in both establishment of first pluripotent lineage and pluripotency maintenance of embryonic stem cells (ESCs) in mice. However, the histone substrates and role of CARM1 in early embryonic development remain largely unknown. Here, we show that CARM1 specifically catalyzes H3R26me2 to promote porcine blastocyst formation. The putative histone substrates of CARM1, including H3R2me2, H3R17me2, and H3R26me2, are present in pig early embryos. The changes of *CARM1* mRNA during early embryogenesis parallel that of H3R26me2. Functional studies using a combinational approach of chemical inhibition and RNA interference (RNAi) showed that catalytic activity inhibition of CARM1 protein or knockdown (KD) of *CARM1* mRNA did not alter the levels of both H3R2me2 and H3R17me2, but significantly reduced H3R26me2 levels in porcine embryos. Furthermore, CARM1 inhibition or KD did not affect embryo development to the 2-cell, 4-cell, 8-cell, and morula stages, but severely compromised blastocyst development. *CARM1* knocked down embryos that developed to the blastocyst stage had fewer total cells, inner cell mass (ICM), and trophectoderm (TE) cells. Mechanistically, single embryo RNA-sequencing analysis revealed that *CARM1* KD altered the transcriptome characterized by downregulation of key genes associated with Hippo and PI3K-AKT signaling pathways. Taken together, these results demonstrate that CARM1 specifically catalyzes H3R26me2 in porcine embryos and participates in blastocyst development.

## Introduction

The blastocyst formation is an important cellular event during preimplantation embryonic development. Concomitantly, the first cell segregation generates pluripotent and differentiating lineages, leading to the formation of ICM and TE in blastocysts (Chazaud and Yamanaka, 2016; White and Plachta, 2020). The first lineage specification is tightly regulated by the restricted expression of master transcription factors (Rossant, 2018) and signaling pathways (Menchero et al., 2017). Epigenetic regulation on the chromatin is also critical for establishing lineage-specific gene expression during early embryogenesis. Accumulating studies showed that methylation on the DNA (Nakanishi et al., 2012) and histone lysine residues (Gao et al., 2010; Liu et al., 2016), and accessible chromatin landscape (Yang et al., 2020) present asymmetrical distribution between ICM and TE in blastocysts and play essential roles in the lineage specification. However, epigenetic regulation of the first lineage specification with histone arginine methylation remains poorly understood.

CARM1 (also known as PRMT4) is recognized as a transcriptional activator and belongs to the family of protein arginine methyltransferase. CARM1 usually mediates asymmetric arginine dimethylation on both histone tails and non-histone proteins (Shishkova et al., 2017; Jarrold and Davies, 2019). It was reported that CARM1 mainly catalyzes the dimethylation at histone H3 arginine 2 (H3R2), 17 (H3R17), and 26 (H3R26) (Schurter et al., 2001). It is worth noting that CARM1 preferentially methylates H3R17 over H3R26 in a cell type specific manner (Jacques et al., 2016). In addition, CARM1 can catalyze H3R17me2 in mouse early embryos (Torres-Padilla et al., 2007), but not in mouse embryonic fibroblasts (Cheng et al., 2020). This implies that CARM1’s option for histone arginine substrates has context-dependent effects. Considering the unique consequences of each methylargine isoform, it is necessary to determine CARM1’s histone substrate under a specific species or cellular context.

At the molecular level, CARM1-mediated histone dimethylation has been implicated in RNA transcription, pre-mRNA splicing, mRNA translation, and genome stability (Lorton and Shechter, 2019). CARM1 is also involved in several cellular processes, including pluripotency maintenance of ESCs (Wu et al., 2009), differentiation of epithelial cells (O’Brien et al., 2010), and spermatogenesis (Bao et al., 2018). In mouse blastocysts, ICM cells express a higher level of CARM1 protein relative to TE cells (Sun et al., 2020). Maternal and zygotic CARM1-deficient embryos could develop into blastocysts that contained fewer ICM cells (White et al., 2016; Xia et al., 2018). Correspondingly, *CARM1* overexpression-induced H3R26me2 is sufficient to contribute blastomere progeny to the ICM lineage in blastocysts (Torres-Padilla et al., 2007). Endogenous CARM1 in mouse embryos suppresses the expression of trophectodermal genes to ensure ICM lineage specification (Parfitt and Zernicka-Goetz, 2010). Intriguingly, a recent study by base editing in mouse embryos showed that CARM1-catalyzed H3R17me2 is required for blastocyst formation (Yang et al., 2019). However, the role of CARM1-mediated H3R26me2 in TE and blastocyst development remains largely unclear.

Given the similarity of reproductive physiology, embryonic developmental timing in pigs to humans (Mordhorst and Prather, 2017; Alberio, 2020), studies in the pig may be informative for understanding the development of human embryos. In the present study, we investigated the role and potential mechanisms of CARM1 in porcine early embryonic development. We found that CARM1 specifically recognizes H3K26me2 in porcine early embryos. Functional studies using RNAi reveal that CARM1-mediated H3R26me2 is indispensable for both lineage specification and blastocyst formation. Single-embryo RNA sequencing analysis demonstrate that CARM1 regulates the expression of key genes required for lineage specification. Our findings provide new insights into the role of CARM1-catalyzed H3R26me2 in porcine blastocyst development.

## Materials and Methods

All reagents used were purchased from Sigma (Sigma-Aldrich, St Louis, MO) unless otherwise stated. Animal experiments were conducted in accordance with the Institutional Animal Care and Use Committee (IACUC) guidelines under current approved protocols at Anhui Agricultural University.

### Preparation of CARM1 Inhibitor

CARM1 inhibitor (Millipore, 217531) was dissolved in DMSO (Sigma, D2650) and stored at −20°C. Embryo culture medium was used to dilute the stock solution to obtain the desired working solution. The same volume of DMSO was added into the medium as a control when the chemical was used.

### Oocyte *in vitro* Maturation

Ovaries were collected from a local slaughterhouse. Follicular fluid was aspirated from antral follicles at 3–6 mm in diameter. Cumulus-oocyte complexes (COCs) were selected under a stereomicroscope. Subsequently, COCs were cultured in one well of 4-well plate containing 400 μL *in vitro* maturation medium for 44 h at 38.5°C, 5% CO_2_ and saturated humidity. Cumulus cells surrounding oocytes was removed using 1 mg/mL hyaluronidase following maturation.

### Parthenogenetic Activation (PA)

Oocytes at metaphase II stage were stimulated using two pulses of direct current (1.56 kV/cm for 80 ms) in activation medium. Subsequently, the activated oocytes were washed three times in the porcine zygote medium (PZM-3) medium and were incubated in the chemically assisted activation medium for 4 h. Then, embryos were cultured in PZM-3 droplets at 38.5°C, 5% CO_2_ and 95% air with saturated humidity.

### *In vitro* Fertilization (IVF)

Oocytes at the metaphase II were washed in the modified Tris-buffered medium (mTBM) containing 2 mg/mL BSA and 2 mM caffeine. The oocytes were incubated in mTBM for 4 h at 38.5°C in 5% CO_2_ in air. Semen of two boars was mixed and centrifuged at 1,900 g for 4 min in DPBS supplemented with 1 mg/mL BSA. Following the removal of supernatant, the sperm concentration was adjusted with mTBM to 1 × 10^6^ sperms/mL. The semen was then added to the mTBM droplets containing oocytes. After co-incubation of oocyte and sperm for 6 h, sperms surrounding oocytes were washed out and presumptive zygotes were cultured in PZM-3 at 38.5 C in 5% CO_2_ in air.

### *In vitro* Transcription

*CARM1-mCherry* mRNA used for microinjection was synthesized *in vitro*. pIVT- *CARM1-mCherry* plasmid containing T7 promoter was linearized by digestion with BspQI. Linearized DNA templates were purified using a DNA clean and concentrator Kit (ZYMO RESEARCH, D4003, Tustin, CA, United States). According to the manufacture’s manual, *in vitro* transcription of *CARM1-mCherry* mRNA was performed through using mMESSAGE MACHINE TM T7 kit (Ambion, AM1344, shanghai, China) and Poly (A) Tailing Kit (Ambion, AM1350, Shanghai, China). Then, mRNA was treated with TURBO DNase to remove the DNA templates and was further purified using MEGAclear Kit (Ambion, AM1908, Shanghai, China). After mRNA was dissolved in RNase-free water, mRNA concentration was determined by a NanoDrop instrument (Thermo Fisher Scientific, Shanghai, China) and was then aliquoted and stored at −80°C.

### Real-Time Quantitative Polymerase Chain Reaction (qPCR)

Total RNA was extracted from oocytes and embryos using RNeasy Mini Kit (Qiagen, 74104). RNA was transcriptionally reversed into cDNA using QuantiTect Reverse Transcription Kit (Qiagen, 205311). The primers used in this study were listed in Supplementary Table 1. The assembly of polymerase chain reaction was prepared in FastStart SYBR Green Master (Roche, 04673514001) and was run on StepOne Plus (Applied Biosystems, Foster, United States). The samples were collected three times and three biological replicates were conducted for each gene. *EF1A1* was used as the internal reference gene. The Cq values were obtained and analyzed using the 2^–ΔΔCt^ method.

### Immunofluorescence Staining

Embryos were fixed in 4% paraformaldehyde solution for 15 min, permeabilized with 1% Triton X-100 for 30 min at room temperature (RT) and then blocked with 2% BSA at RT for 1 h. Samples were incubated in solution containing primary antibodies overnight at 4°C. Following washing, samples were incubated for 1 h in solution containing secondary antibodies in the dark at 37°C. Following washing, samples were counterstained using 4, 6-diamidino-2-phenylindole dihydrochloride or propidium iodide for 10 min and were then loaded onto glass slides. Finally, samples were imaged using laser scanning confocal microscopy (Olympus, Japan). Information regarding primary and secondary antibodies used was provided in Supplementary Table 2.

### Microinjection

siRNA species was designed to target three different sites of the porcine *CARM1* coding region (GenePharma, Shanghai, China). Microinjection was performed in a T2 (TCM199 with 2% FBS) medium containing 7.5 μg/mL Cytochalasin B on an inverted microscope (Olympus, Japan). Approximately 10 pl of siRNA solution (50 μM) was microinjected into the cytoplasm of MII oocytes. Embryos were cultured in PZM-3 medium for 7 days.

### Single-Embryo RNA Sequencing

Single embryo at day 5 (non-injected and *CARM1*-siRNA injected embryos) was collected for RNA-seq analysis. RNA was extracted using the RNeasy Mini Kit (Qiagen, 74104). Pre-amplified cDNA was fragmented using Fragmentase (NEB, M0348S) via the incubation at 37°C for 20 min. cDNA libraries were constructed by TruSeq Nano DNA LT Library Preparation Kit (FC-121-4001). Then, the libraries were sequenced on the Illumina HiSeq 2500 instrument (LC-Sciences, Hangzhou, China). The reads were mapped to the pig reference genome. Differential gene expression between non-injectedand *CARM1* siRNA injected embryos was determined using Cufflinks. The threshold for significance was a false discovery rate ≤0.05 and a fold expression change ≥2. GO analysis was performed using DAVID Bioinformatics Resources 6.8. RNA-seq data are presented in Supplementary Tables 3–5.

### Statistical Analysis

Statistical analyses were performed using one-way ANOVA or Student’s *t*-test (SPSS 17.0). All experiments were carried out at least three times and were presented as mean ± standard error of mean (mean ± S.E.M). *P* < 0.05 was considered to be statistically significant.

## Results

### Characterization of *CARM1* mRNA and Potential Histone Substrates in Early Embryos

**FIGURE 1:**
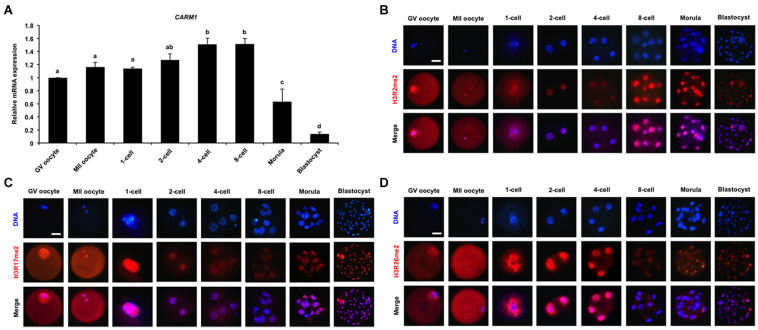
Dynamics of CARM1 mRNA and putative histone substrates in early embryos. **(A)** Expression of *CARM1* mRNA in porcine early embryos. Relative abundance of *CARM1* mRNA was determined by qPCR. Data were normalized to the reference gene (*EF1*α*1*) and the data from GV oocytes were set as 1. GV, germinal vesicle; MII, metaphase II. Data are shown as mean ± S.E.M and different letters on the bars indicate significant differences (*P* < 0.05). Changes of H3R2me2 **(B)**, H3R17me2 **(C)**, H3R26me2 **(D)** levels in early embryonic development. Embryos were stained for H3R2me2, H3R17me2, H3R26me2 (red), and DNA (blue). Representative images obtained by confocal microscopy are shown. The experiment was independently repeated three times with at least 30 embryos per group. Scale bar: 50 μm.

To quantify the relative abundance of *CARM1* mRNA in early embryos, qPCR was performed to determine the expression of *CARM1* mRNA. The results revealed that *CARM1* is expressed in oocytes and embryos, but the expression levels of *CARM1* mRNA are significantly higher in embryos at the 4-cell and 8-cell stages compared to oocytes, morulae, and blastocysts (Figure 1A) (*P* < 0.05). Furthermore, immunofluorescence staining was performed to determine the localization of CARM1’s putative histone substrates including H3R2me2, H3R17me2, and H3R26me2 in porcine embryos. The specificity of the arginine dimethylation antibodies in porcine blastocysts had been verified before undergoing immunostaining experiment (Supplementary Figure 1A). Immunofluorescence analysis showed that the dimethylation modifications of three histone arginine residues simultaneously localize at the cytoplasm and nuclei of both GV and MII oocytes whereas they are present in nuclei throughout early embryonic development (Figures 1B–D). Additionally, the dimethylation modifications of three histone arginine residues are also present in both the ICM and TE cells of blastocysts (Figures 1B–D). Therefore, these results indicate that *CARM1* mRNA and its putative dimethylation modifications of histone arginine residues are dynamically present in early embryonic development.

### Chemical Inhibition of CARM1 Activity Blocks Blastocyst Formation and Reduces H3R26me2 Levels in Early Embryos

**FIGURE 2:**
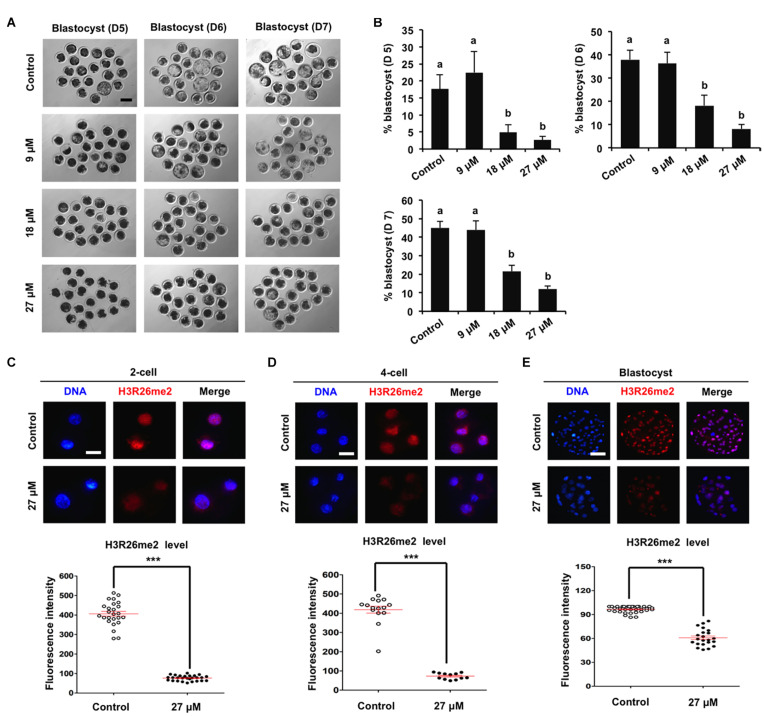
Effect of CARM1 inhibition on blastocyst development and H3R26me2 levels in embryos. **(A)** Representative images of blastocysts at days 5, 6, and 7 from control and treatment groups. Scale bar: 100 μm. **(B)** Effect of treatment of CARM1 inhibitor with different concentrations on blastocyst rates. Effect of CARM1 inhibition on H3R26me2 levels in 2-cell **(C)**, 4-cell **(D)**, and blastocysts **(E)**. Embryos at different stages were stained for H3R26me2 (red) and DNA (blue). The experiment was independently repeated three times with at least 12 embryos per group. Scale bar: 50 μm. All data are shown as mean ± S.E.M and different letters on the bars or asterisks indicate significant differences (*P* < 0.05).

To determine the specific histone arginine substrates of CARM1 in porcine embryos, a chemical inhibitor with different concentrations (9, 18, and 27 μM) was used to treat embryos. The developmental results revealed that treatment of 9 μM CARM1 inhibitor did not affect the developmental rates of early embryos (Figures 2A,B and Supplementary Figures 2A,B). Treatment of 18 μM CARM1 inhibitor partially hindered the development of 2-cell to morula whereas it significantly reduced the blastocyst rates (Figures 2A,B and Supplementary Figures 2A,B) (*P* < 0.05). Treatment of 27 μM CARM1 inhibitor significantly blocked the development of 2-cell to blastocyst stages (Figures 2A,B and Supplementary Figures 2A,B) (*P* < 0.05). Considering the developmental phenotypes of embryos exposed to different concentrations of CARM1 inhibitor, the dosage of 27 μM was used in the subsequent experiments. To further determine the levels of H3R2me2, H3R17me2, and H3R26me2, immunofluorescence staining was performed in embryos at the 2-cell, 4-cell, and blastocyst stages. The results showed that CARM1 inhibition did not affect H3R2me2 and H3R17me2 levels, but significantly reduced H3R26me2 levels in embryos at the indicated stages (Figures 2C–E and Supplementary Figures 2C,D) (*P* < 0.05). Therefore, these data demonstrate that CARM1 activity is required for blastocyst formation and the generation of H3R26me2 modification in porcine embryos.

### siRNA Injection Efficiently Attenuates Expression of CARM1 mRNA and Protein and Specifically Decreases H3R26me2 Levels in Early Embryos

**FIGURE 3:**
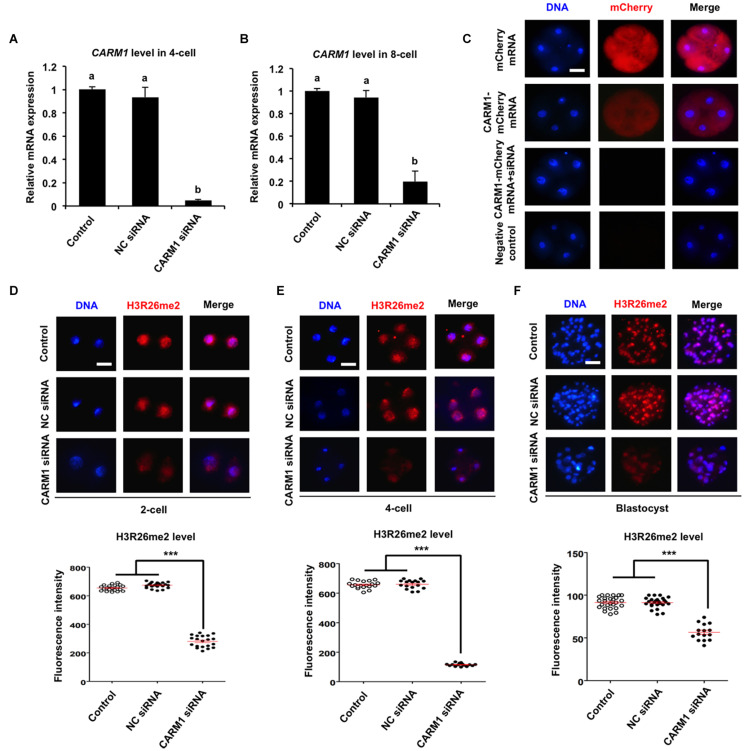
Effect of siRNA injection on CARM1 expression and H3R26me2 levels in embryos. Expression levels of *CARM1* mRNA in 4-cell **(A)** and 8-cell **(B)** from control, NC siRNA injection, and *CARM1* siRNA injection were determined by qPCR. NC, negative control. **(C)** Expression of CARM1 protein in 4-cell embryos. Embryos from the indicated groups were imaged for mCherry (red) and DNA (blue) by confocal microscopy and the representative images are shown. The experiment was independently repeated three times with at least 20 embryos per stage. Scale bar: 50 μm. Effect of siRNA injection on H3R26me2 levels in 2-cell **(D)**, 4-cell **(E)**, and blastocysts **(F)**. Embryos at different stages were stained for H3R26me2 (red) and DNA (blue). The experiment was independently repeated three times with at least 15 embryos per group. Scale bar: 50 μm. All data are shown as mean ± S.E.M and different letters on the bars or asterisks indicate significant differences (*P* < 0.05).

To further confirm whether CARM1 protein specifically recognizes the H3R26 residue in porcine embryos, RNAi approach was used to delete the CARM1 protein. MII oocytes were microinjected with *CARM1* siRNA or negative control (NC) siRNA. Uninjected oocytes served as control groups. A subset of embryos at the 4-cell and 8-cell stage was subject to qPCR to detect the expression levels of *CARM1* mRNA. The results revealed that siRNA injection significantly reduced the levels of *CARM1* mRNA in embryos at the 4-cell (Figure 3A) and 8-cell (Figure 3B) stage compared to the control groups (*P* < 0.05). No differences in expression levels of *CARM1* mRNA were observed between NC group and uninjected group (Figures 3A,B). There are not available for porcine specific CARM1 antibodies so that we could not directly evaluate the levels of CARM1 protein in embryos. Instead, we constructed the fusion expression vector of *CARM1-mCherry* and synthesized *in vitro* the fusion mRNA. Both *CARM1-mCherry* mRNA and siRNA was injected into oocytes and served as the experimental group, uninjected, *mCherry* mRNA or *CARM1-mCherry*mRNA injection served as the control groups. Fluorescence intensity analysis showed that *CARM1* siRNA injection significantly reduced the levels of CARM1-mCherry protein at the 4-cell stage compared to the control groups (Figure 3C) (*P* < 0.05). Next, immunofluorescence staining was performed to evaluate whether *CARM1* KD affected the levels of H3R2me2, H3R17me2, and H3R26me2 in embryos. As shown in Supplementary Figures 3A,B, *CARM1* KD did not alter the levels of both H3R2me2 and H3R17me2 in embryos at the 2-cell, 4-cell, and blastocyst stage. However, *CARM1* KD significantly decreased H3R26me2 levels in embryos at the 2-cell (Figure 3D), 4-cell (Figure 3E), and blastocyst (Figure 3F) stage compared to the control groups (*P* < 0.05). Collectively, these results document that CARM1 protein specifically catalyzes H3R26me2 in porcine early embryos.

### *CARM1* Knockdown Impedes Blastocyst Formation and Disrupts Normal Lineage Allocation

**FIGURE 4:**
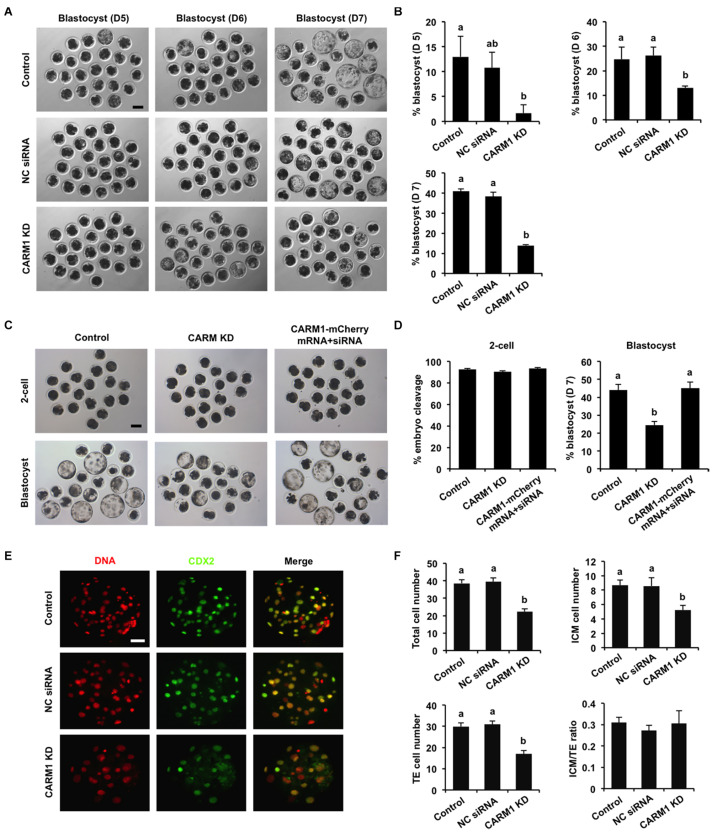
Effect of *CARM1* knockdown on blastocyst development and lineage allocation. **(A)** Representative images of blastocysts from different stages at days 5, 6, and 7. Scale bar: 100 μm. **(B)** Analysis of blastocyst rates at days 5, 6, and 7. **(C)** Representative images of 2-cell embryos and blastocysts. Scale bar: 100 μm. **(D)** Analysis of cleavage and blastocyst rates. **(E)** Representative fluorescence images of blastocysts. Embryos at different groups were stained for CDX2 (green) and DNA (red). The experiment was independently repeated three times with at least 20 blastocysts per group. Scale bar: 50 μm. **(F)** Analysis of lineage allocation in blastocysts. The numbers of total cells, ICM cells, TE cells, and the ratio of ICM cells to TE cells were recorded and subject to statistical analysis. ICM: inner cell mass; TE: trophectoderm. All data are shown as mean ± S.E.M and different letters on the bars indicate significant differences (*P* < 0.05).

To elucidate whether *CARM1* KD affected embryo development, the developmental rates of PA embryos at each stage were subject to statistical analysis. We found that *CARM1* KD had no effect on embryo development to 2-cell, 4-cell, 8-cell, and morula stage (Supplementary Figures 3C,D), but reduced the blastocyst rates (Days 5–7) compared to the control groups (Figures 4A,B) (*P* < 0.05). Additionally, we did not observe differences in the rates of embryo development between NC siRNA injected and uninjected control groups (Figures 4A,B and Supplementary Figures 3C,D). To confirm the specificity of *CARM1* KD, rescue experiments were performed by coinjection of *CARM1*-mCherry mRNA and *CARM1* siRNA in oocytes. The results revealed that coinjection of *CARM1*-mCherry mRNA and *CARM1* siRNA could restore blastocyst formation of *CARM1* KD embryos (Figures 4C,D), demonstrating a specific role of CARM1 in early embryonic development. To determine whether *CARM1* KD impaired lineage allocation in blastocysts, embryos were stained with a CDX2 antibody to determine the TE cell number (Figure 4E). The number of ICM cells was indirectly determined by subtracting the TE number from the total cell number. The results showed that *CARM1* KD resulted in a significant reduction in the number of total cells, ICM and TE cells (Figure 4F) (*P* < 0.05). However, the ratio of ICM cells to TE cells in the *CARM1* KD blastocysts was similar to that in the control groups (Figure 4F). Similarly, *CARM1* KD did not impair the development of IVF embryos before the morula stage (data not shown), while *CARM1* KD led to a significant reduction in both embryos that developed to blastocyst stage (days 5–7) (Supplementary Figures 4A,B) and lineage cells of IVF blastocysts (Supplementary Figures 4C,D) (*P* < 0.05). Hence, these results indicate that CARM1 is indispensable for blastocyst formation and normal lineage allocation.

### *CARM1* Knockdown Alters the Global Transcriptome in Early Embryos

**FIGURE 5:**
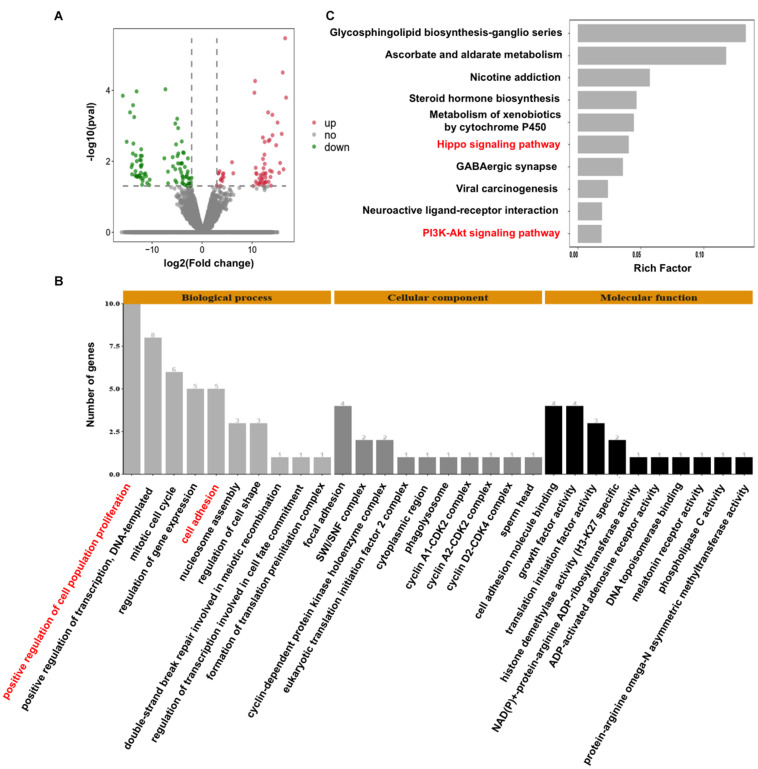
Effect of *CARM1* knockdown on the transcriptome in embryos. **(A)** Volcano plot displaying differentially expressed genes in embryos from control and *CARM1* KD groups. Green spots denote the number of downregulated genes; red spots indicate the number of upregulated genes. **(B)** GO analysis showing the most enriched functional categories enriched by the differentially expressed genes. **(C)** KEGG analysis showing the top 10 signaling pathways enriched by the differentially expressed genes.

To decipher the molecular mechanisms of CARM1 regulating embryo deve0lopment, single-embryo RNA sequencing was utilized to characterize tanscriptomic changes in *CARM1* KD embryos. To validate the single-embryo RNA sequencing data, the expression levels of 6 differentially expressed genes (DEGs) (3 downregulated and 3 upregulated genes), namely *ID2*, *CCND2*, *CARM1*, *PLK5*, *PPP2R2C*, and *CCNA1*, were analyzed by qPCR. The expression patterns of these selected genes are highly consistent with the treads obtained from RNA sequencing data (Supplementary Figure 5), suggesting the robustness of the results obtained by RNA sequencing. We identified a total of 154 DEGs between *CARM1* KD and uninjected control groups, of which there were 89 down-regulated genes and 65 up-regulated genes, respectively (Figure 5A and Supplementary Table 3). Furthermore, GO analysis was performed to annotate the potential function of the DEGs in embryo development. The DEGs were classified into three main categories (biological process, cellular component, and molecular function) according to the GO database. The top-ranking 10 biological processes, 10 cellular components, and 10 molecular functions involved in each GO term were provided (Figure 5B and Supplementary Table 4) (*P* < 0.05), such as positive regulation of cell population proliferation (10 genes, e.g., *AREG*, *CCNA1*, *FGF19*, *CARM1*, *CCND2*), negative regulation of epithelial cell proliferation (4 genes, e.g., *WNT10B*, *GPC3*), cell adhesion (4 genes, e.g., *ADAM8*, *TGFBI*). Lastly, KEGG analysis revealed that the DEGs were mainly enriched in 10 significant pathways (Figure 5C and Supplementary Table 5) (*P* < 0.05), such as Hippo signaling pathway (6 genes, e.g., *WNT10B*, *ID2*, *PPP2R2C*, *AREG*, *ITGB2*, *CCND2*), PI3K-Akt signaling pathway (6 genes, e.g., *AREG*, *FGF19*, *EFNA4*, *CCND2*, *CCNA1*). Altogether, these data demonstrate that CARM1 regulates the expression of key genes important for blastocyst formation.

## Discussion

Although regulatory mechanisms of CARM1-mediated H3R26me2 underlying the ICM lineage specification have been well studied (Parfitt and Zernicka-Goetz, 2010; White et al., 2016; Xia et al., 2018), the role of CARM1-catalyzed histone arginine dimethylation in the TE lineage specification and blastocyst development remains largely unknown. Here, we show that CARM1 specifically catalyzes H3R26me2, but not H3R2me2 and H3R17me2 in porcine early embryos. CARM1-mediated H3R26me2 facilitates lineage specification and blastocyst formation. Mechanistically, CARM1 regulates the expression of multiple genes required for cell proliferation and lineage specification. Therefore, our data demonstrate that CARM1-mediated H3R26me2 participates in porcine blastocyst formation.

Histone arginine methylation encompasses diverse isoforms at different residues and each methylation isoform presents unique function in a specific biological process (Kirmizis et al., 2007; Torres-Padilla et al., 2007). It is well recognized that CARM1 can catalyze H3R2me2, H3R17me2, and H3R26me2 in a specific cell type (Schurter et al., 2001), but the option of CARM1 for histone substrates frequently has context-dependent effects (Price and Hevel, 2020). Studies showed that CARM1 can simultaneously catalyze the dimethylation at R2, R17, and R26 in mouse early embryos (Torres-Padilla et al., 2007), but it does not recognize the H3R17me2 substrate in mouse embryonic fibroblasts (Cheng et al., 2020). In this study, we found that CARM1 specifically catalyzes H3R26me2, but not H3R2me2 and H3R17me2 in porcine early embryos. The discrepancy in the recognition of CARM1 substrates in early embryos could be due to species differences. The development of early embryos exhibits a unique feature between mice and pigs (Alberio, 2020), which might cause a different requirement for CARM1’s histone substrates. Since CARM1 also catalyzes non-histone proteins in some cell types (Shishkova et al., 2017), we could not rule out the possibility that CARM1 targets other arginine substrates in porcine early embryos.

Accumulating evidences indicated that epigenetic regulation plays a critical role in the lineage specification of mammalian blastocyst (Paul and Knott, 2014; Fu et al., 2020). The results in this study showed that *CARM1* KD led to a significant reduction in the number of ICM and TE cells, suggesting that CARM1 regulates lineage allocation in porcine blastocysts. In mouse preimplantation embryos, CARM1-mediated H3R26me2 limits trophectodermal features to bias blastomeres toward ICM lineage (Torres-Padilla et al., 2007). Similarly, our transcriptomic analysis revealed that CARM1 regulates Hippo signaling pathway in porcine embryos. Disruption of Hippo signaling leads to the misspecification of the ICM lineage in several species (Nishioka et al., 2009; Negron-Perez and Hansen, 2018; Cao et al., 2019). Thus, we speculate that CARM1 may activate Hippo signaling to facilitate ICM lineage specification in pigs. On the other hand, CARM1 is required for differentiation of epithelial cells (O’Brien et al., 2010) and enforced expression of CARM1 promoted *CDX2* transcription and increased TE cell number in murine cloned blastocysts (Bang et al., 2018). It is thus likely that CARM1 elevates the expression of trophectoderm genes to promote TE lineage specification. Altogether, these results lead us to reason that CARM1 may exert differential mechanisms to allow the first lineage specification in porcine blastocysts.

Whether CARM1 is essential for blastocyst formation in mice has not reached consistent conclusions (White et al., 2016; Yang et al., 2019), but our data document that CARM1 is indispensable for porcine blastocyst development. A number of signaling pathways have been shown to be essential for blastocyst formation in mammals (Maekawa et al., 2005; Nakasato et al., 2006; Jiao et al., 2020). In this study, single-embryo transcriptomic analysis revealed that CARM1 is implicated in both Hippo and PI3K-AKT signaling pathways, in which amphiregulin (*AREG)* was downregulated in *CARM1* KD embryos. Previous studies established that AREG peptide supplementation elevated blastocyst rates in mice (Richani et al., 2013) and pigs (Prochazka et al., 2011). In addition, inhibition of Akt activity blocked murine blastocyst formation (Riley et al., 2005), and inactivation of PI3K signaling also prevented porcine blastocyst development (Jiao et al., 2020). Together, these data demonstrate that CARM1 in porcine embryos facilitates blastocyst formation via modulating the expression of key genes involved in signaling pathways. Future chromatin immunoprecipitation (ChIP) studies in pig embryos are warranted to confirm whether CARM1 regulation on these genes depends on H3R26me2.

## Conclusion

In conclusion, our findings demonstrate that CARM1-mediated H3R26me2 is required for blastocyst development in pigs. Our results may provide new insights into the regulatory mechanism of CARM1-catalyzed H3R26me2 in porcine blastocyst development. These findings will be instrumental in developing novel strategies to improve developmental competence of porcine *in vitro* production embryos.

## Data Availability Statement

The data presented in the study are deposited in the GEO repository, accession number (GSE173965).

## Ethics Statement

Animal experiments were conducted in accordance with the Institutional Animal Care and Use Committee (IACUC) guidelines under current approved protocols at Anhui Agricultural University.

## Author Contributions

ZC and YZ designed the research and wrote the manuscript. XT, HY, NZ, XZ, MZ, XW, QL, and YY performed the experiments. XT, HY, NZ, and XZ analyzed the data. YM, TY, and YL revised the manuscript. All authors read and approved the final version of manuscript.

## Conflict of Interest

The authors declare that the research was conducted in the absence of any commercial or financial relationships that could be construed as a potential conflict of interest.

## Supplementary Material

Supplementary Figure 1 Verification of the specificity of H3R2me2, H3R17me2, and H3R26me2 antibody. H3R2me2, H3R17me2, and H3R26me2 antibody was tested on porcine blastocysts. The secondary antibody was replaced with blocking buffer to serve as a negative control. Representative images obtained by confocal microscopy are shown. Scale bar: 50 μm.

Supplementary Figure 2 Effect of CARM1 inhibition on cleavage-stage embryonic development, H3R2me2, and H3R17me2 levels in embryos. **(A)** Representative images of embryos at different stages. Scale bar: 100 μm. **(B)** Developmental rates of 2-cell, 4-cell, 8-cell, and morula in the control and the *CARM1* inhibition groups. Effect of CARM1 inhibition on H3R2me2 **(C)** and H3R17me2 **(D)** levels in 2-cell, 4-cell, and blastocysts. Embryos at different stages were stained for H3R2me2, H3R17me2 (red), and DNA (blue). The experiment was independently repeated three times with at least 15 embryos per group. Scale bar: 50 μm. All data are shown as mean ± S.E.M and different letters on the bars indicate significant differences (*P* < 0.05).

Supplementary Figure 3 Effect of *CARM1* knockdown on H3R2me2 and H3R17me2 levels in embryos, and cleavage-stage embryonic development. Effect of *CARM1* KD on H3R2me2 **(A)** and H3R17me2 **(B)** levels in 2-cell, 4-cell, and blastocysts. Embryos at different stages were stained for H3R2me2, H3R17me2 (red), and DNA (blue). The experiment was independently repeated three times with at least 11 embryos per group. Scale bar: 50 μm. **(C)** Representative images of embryos at different stages. Scale bar: 100 μm. **(D)** Developmental rates of 2-cell, 4-cell, 8-cell, and morula in the control and the *CARM1* KD groups. All data are expressed as mean ± S.E.M and different letters on the bars indicate significant differences (*P* < 0.05).

Supplementary Figure 4 Effect of *CARM1* knockdown on blastocyst formation and lineage allocation of *in vitro* fertilized embryos. **(A)** Representative images of IVF blastocysts from different stages at days 5, 6, and 7. Scale bar: 100 μm. **(B)** Percentage of blastocyst rates at days 5, 6, and 7. **(C)** Representative fluorescence images of IVF blastocysts. Embryos at different groups were stained for CDX2 (green) and DNA (blue). The experiment was independently repeated three times with at least 20 embryos per group. Scale bar: 50 μm. **(D)** Analysis of lineage allocation in IVF blastocysts. The numbers of total cells, ICM cells, TE cells, and the ratio of ICM cells to TE cells were recorded and subject to statistical analysis. ICM: inner cell mass; TE: trophectoderm. All data are expressed as mean ± S.E.M and different letters on the bars indicate significant differences (*P* < 0.05).

Supplementary Figure 5 Validation of single-embryo RNA sequencing data by qPCR. Relative abundance of six differentially expressed genes was determined by qPCR. The experiment was independently repeated three times. Data were normalized to the housekeeping gene (*EF1*α*1*) and the data from control group were set as 1. Data are shown as mean ± S.E.M and different letters on the bars indicate significant differences (*P* < 0.05).
