# Supplementary figures and images for: Histone Arginine Methyltransferase CARM1-Mediated H3R26me2 Is Essential for Morula-to-Blastocyst Transition in Pigs
Source: Front Cell Dev Biol. 2021 Jun 2;9:678282. doi: 10.3389/fcell.2021.678282 (PMC8206646; doi:10.3389/fcell.2021.678282)

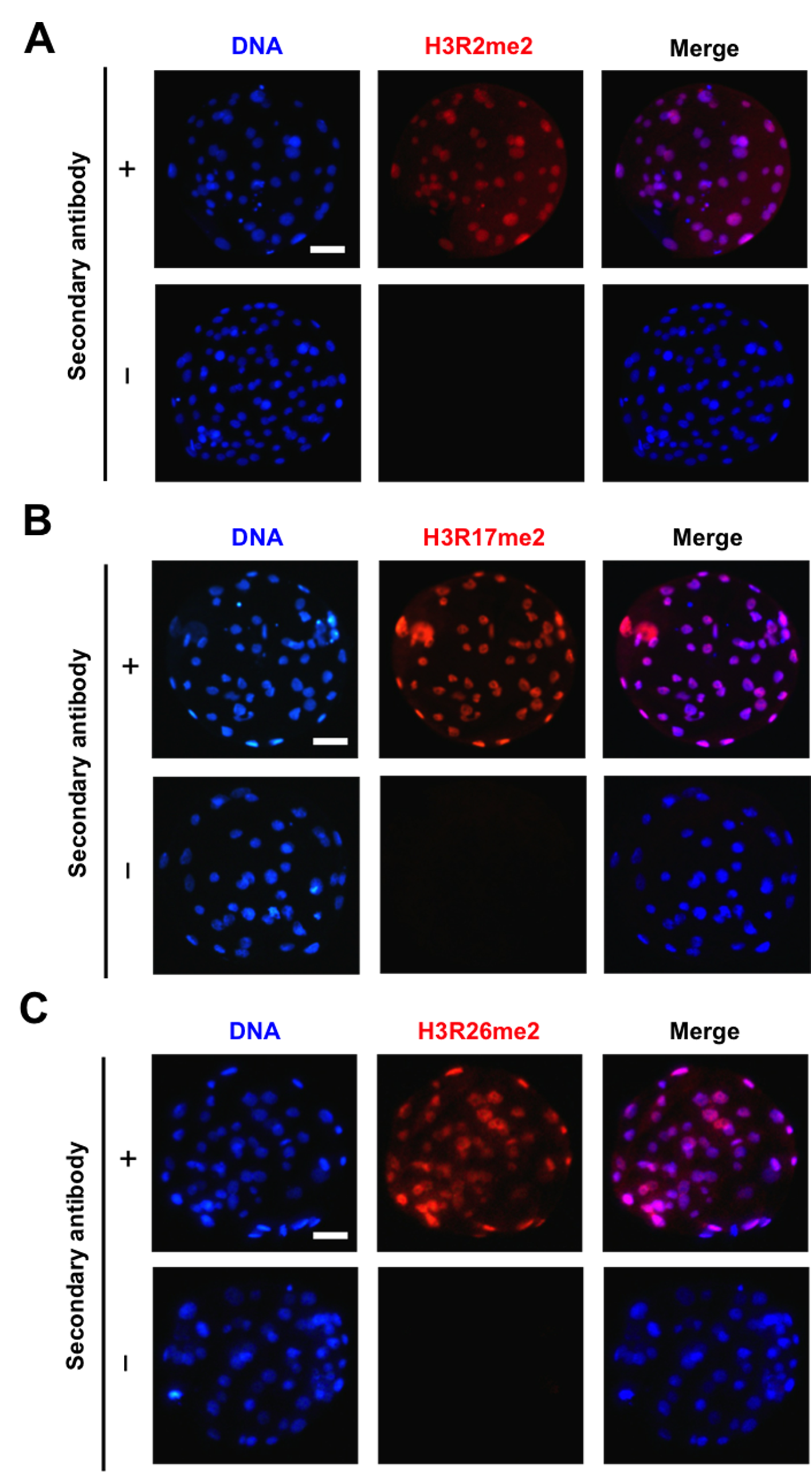

Supplement: Supplementary Figure 1 — Verification of the specificity of H3R2me2, H3R17me2, and H3R26me2 antibody. H3R2me2, H3R17me2, and H3R26me2 antibody was tested on porcine blastocysts. The secondary antibody was replaced with blocking buffer to serve as a negative control. Representative images obtained by confocal microscopy are shown. Scale bar: 50 μm. [file Image_1.tif]

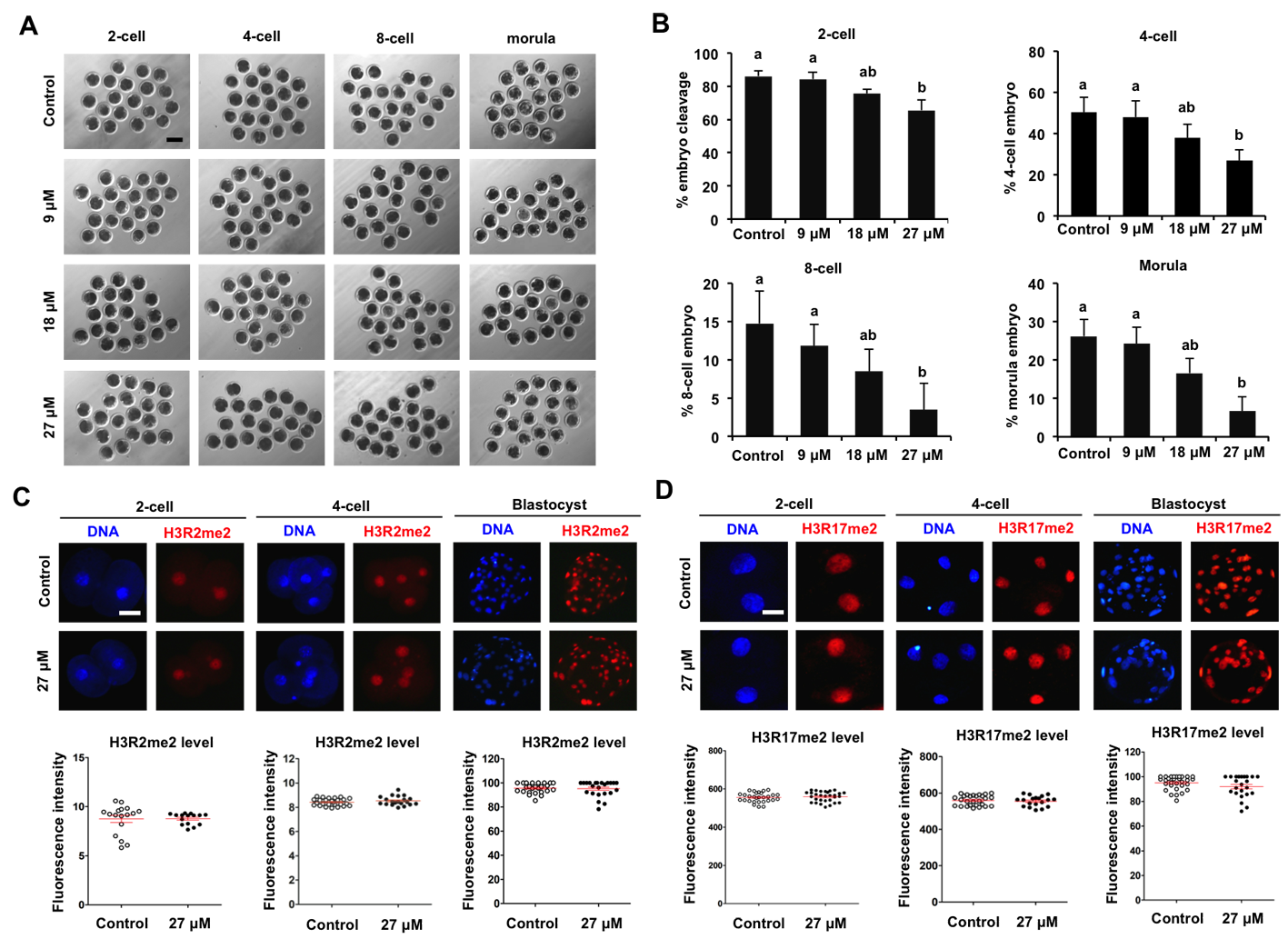

Supplement: Supplementary Figure 2 — Effect of CARM1 inhibition on cleavage-stage embryonic development, H3R2me2, and H3R17me2 levels in embryos. (A) Representative images of embryos at different stages. Scale bar: 100 μm. (B) Developmental rates of 2-cell, 4-cell, 8-cell, and morula in the control and the CARM1 inhibition groups. Effect of CARM1 inhibition on H3R2me2 (C) and H3R17me2 (D) levels in 2-cell, 4-cell, and blastocysts. Embryos at different stages were stained for H3R2me2, H3R17me2 (red), and DNA (blue). The experiment was independently repeated three times with at least 15 embryos per group. Scale bar: 50 μm. All data are shown as mean ± S.E.M and different letters on the bars indicate significant differences (P < 0.05). [file Image_2.tif]

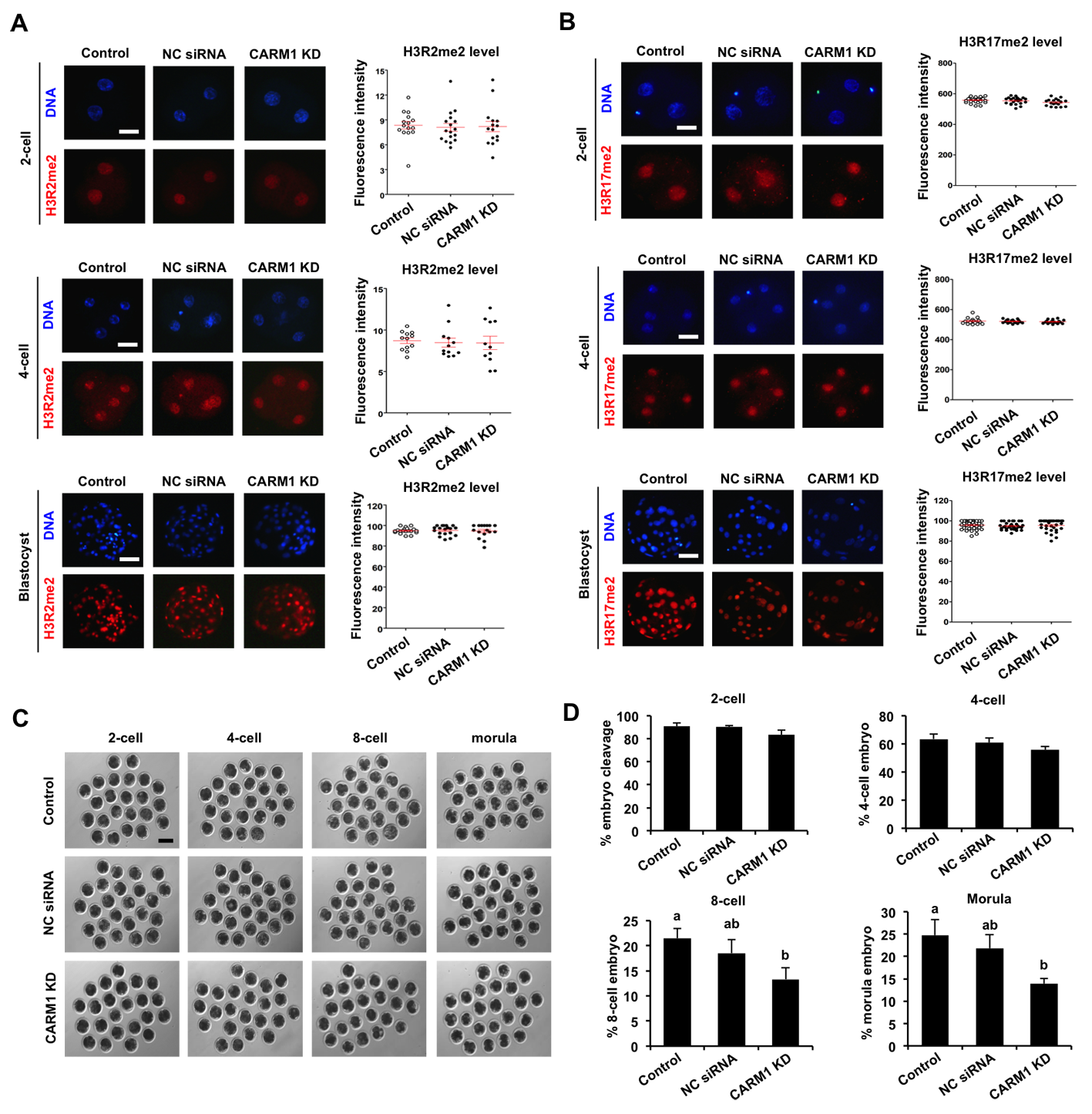

Supplement: Supplementary Figure 3 — Effect of CARM1 knockdown on H3R2me2 and H3R17me2 levels in embryos, and cleavage-stage embryonic development. Effect of CARM1 KD on H3R2me2 (A) and H3R17me2 (B) levels in 2-cell, 4-cell, and blastocysts. Embryos at different stages were stained for H3R2me2, H3R17me2 (red), and DNA (blue). The experiment was independently repeated three times with at least 11 embryos per group. Scale bar: 50 μm. (C) Representative images of embryos at different stages. Scale bar: 100 μm. (D) Developmental rates of 2-cell, 4-cell, 8-cell, and morula in the control and the CARM1 KD groups. All data are expressed as mean ± S.E.M and different letters on the bars indicate significant differences (P < 0.05). [file Image_3.tif]

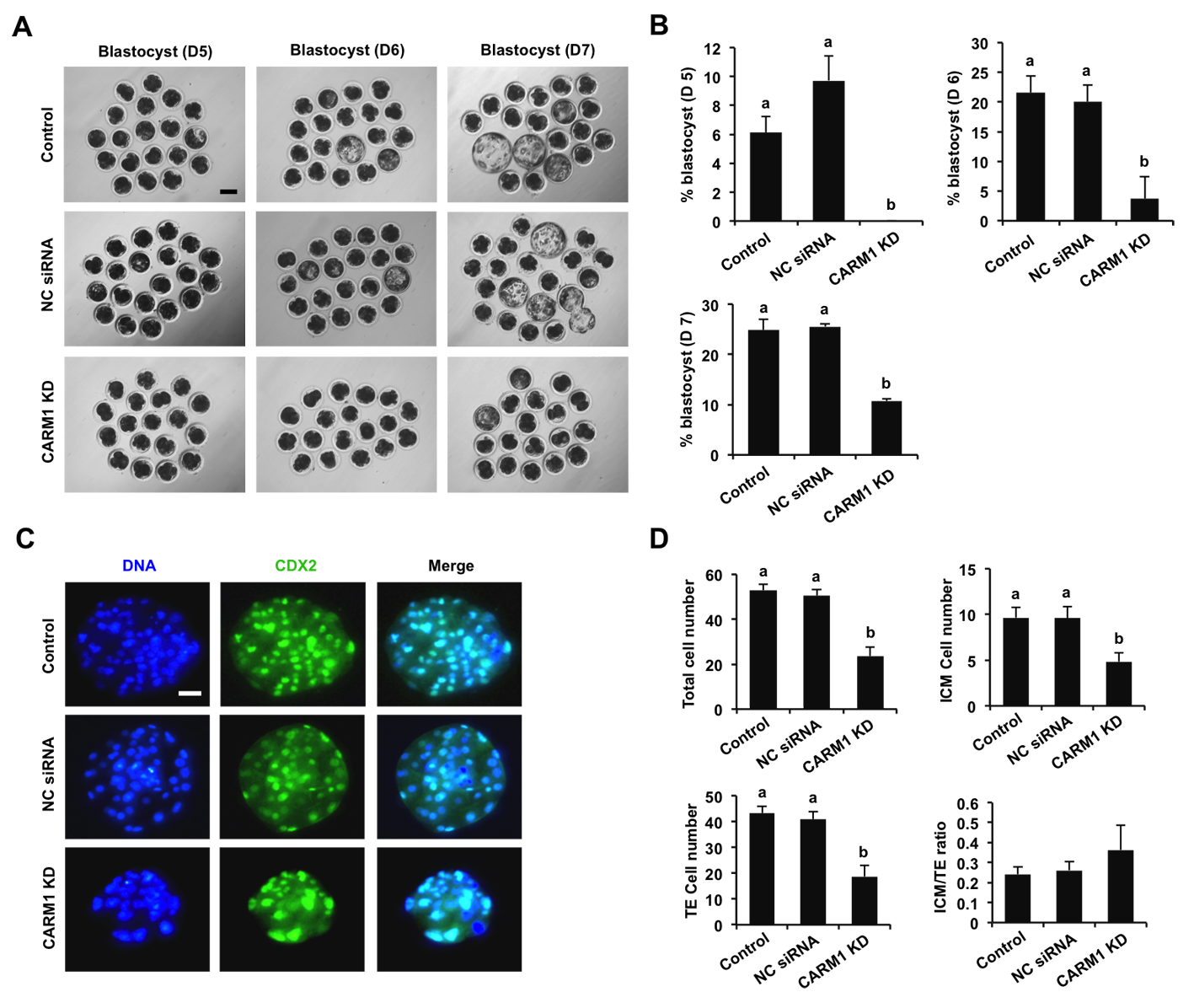

Supplement: Supplementary Figure 4 — Effect of CARM1 knockdown on blastocyst formation and lineage allocation of in vitro fertilized embryos. (A) Representative images of IVF blastocysts from different stages at days 5, 6, and 7. Scale bar: 100 μm. (B) Percentage of blastocyst rates at days 5, 6, and 7. (C) Representative fluorescence images of IVF blastocysts. Embryos at different groups were stained for CDX2 (green) and DNA (blue). The experiment was independently repeated three times with at least 20 embryos per group. Scale bar: 50 μm. (D) Analysis of lineage allocation in IVF blastocysts. The numbers of total cells, ICM cells, TE cells, and the ratio of ICM cells to TE cells were recorded and subject to statistical analysis. ICM: inner cell mass; TE: trophectoderm. All data are expressed as mean ± S.E.M and different letters on the bars indicate significant differences (P < 0.05). [file Image_4.tif]

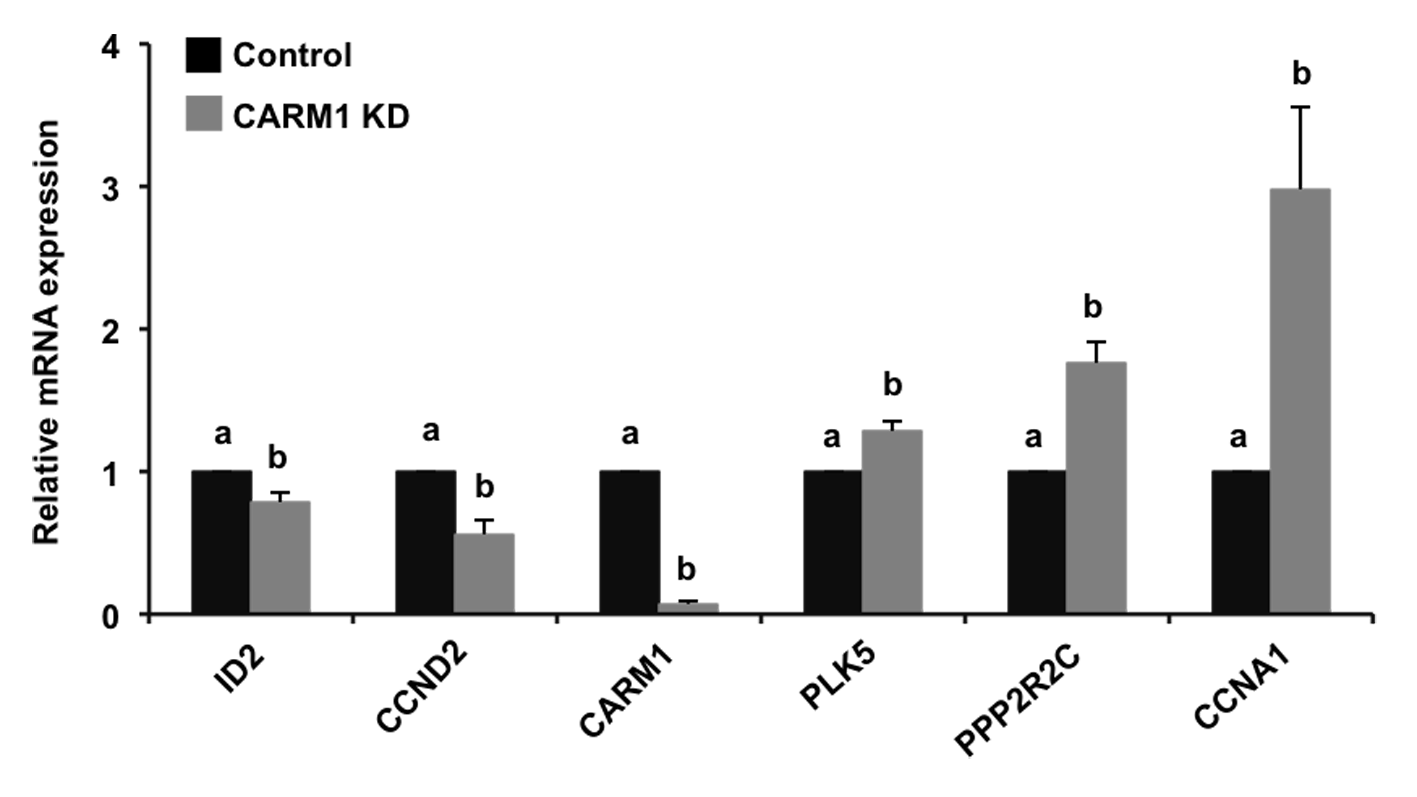

Supplement: Supplementary Figure 5 — Validation of single-embryo RNA sequencing data by qPCR. Relative abundance of six differentially expressed genes was determined by qPCR. The experiment was independently repeated three times. Data were normalized to the housekeeping gene (EF1α1) and the data from control group were set as 1. Data are shown as mean ± S.E.M and different letters on the bars indicate significant differences (P < 0.05). [file Image_5.tif]
